# Modification of phosphoinositides by the *Shigella* effector IpgD during host cell infection

**DOI:** 10.3389/fcimb.2022.1012533

**Published:** 2022-10-27

**Authors:** Guy Tran Van Nhieu, Patricia Latour-Lambert, Jost Enninga

**Affiliations:** ^1^ Institute for Integrative Biology of the Cell – Centre National de la Recherche Scientifique (CNRS) UMR9198 - Institut National de la Santé et de la Recherche Médicale (Inserm) U1280, Team Calcium Signaling and Microbial Infections, Gif-sur-Yvette, France; ^2^ Institut Pasteur, Unité Dynamique des interactions hôtes-pathogènes and Centre National de la Recherche Scientifique (CNRS) UMR3691, Université de Paris Cité, Paris, France

**Keywords:** *Shigella*, IpgD, phosphatidyl-inositol polyphosphate, phosphatase, invasion, dissemination, inflammation, phosphotransferase

## Abstract

*Shigella*, the causative agent of bacillary dysentery, subvert cytoskeletal and trafficking processes to invade and replicate in epithelial cells using an arsenal of bacterial effectors translocated through a type III secretion system. Here, we review the various roles of the type III effector IpgD, initially characterized as phosphatidylinositol 4,5 bisphosphate (PI4,5P_2_) 4-phosphatase. By decreasing PI4,5P_2_ levels, IpgD triggers the disassembly of cortical actin filaments required for bacterial invasion and cell migration. PI5P produced by IpgD further stimulates signaling pathways regulating cell survival, macropinosome formation, endosomal trafficking and dampening of immune responses. Recently, IpgD was also found to exhibit phosphotransferase activity leading to PI3,4P_2_ synthesis adding a new flavor to this multipotent bacterial enzyme. The substrate of IpgD, PI4,5P_2_ is also the main substrate hydrolyzed by endogenous phospholipases C to produce inositoltriphosphate (InsP_3_), a major Ca^2+^ second messenger. Hence, beyond the repertoire of effects associated with the direct diversion of phoshoinositides, IpgD indirectly down-regulates InsP_3_-mediated Ca^2+^ release by limiting InsP_3_ production. Furthermore, IpgD controls the intracellular lifestyle of *Shigella* promoting Rab8/11 -dependent recruitment of the exocyst at macropinosomes to remove damaged vacuolar membrane remnants and promote bacterial cytosolic escape. IpgD thus emerges as a key bacterial effector for the remodeling of host cell membranes.

## Introduction

Invasive pathogens subvert cytoskeletal processes to promote their internalization by normally non-phagocytic cells. Once inside a targeted host cell, the pathogens may reside inside a membrane-bound bacterial-containing vacuole (BCV) or escape from this compartment. Diversion of endocytic processes has been mostly described for pathogens living inside BCVs. Recent evidence indicates that freely replicating bacteria within the cell cytosol, such as *Shigella*, the causative agent of bacillary dysentery, also divert host trafficking to promote vacuolar escape. Because of their central role in cytoskeletal reorganization and endosomal trafficking, phosphoinositides are targeted by virulence factors from a variety of invasive bacteria (Payrastre et al., 2012; Pizarro-Cerdá et al., 2015). Among these, type III secretion system (T3SS) effectors that are injected into the host cell cytosol are of particular interest. They alter general cellular signaling pathways, as well as exhibiting localized activities at bacterial entry sites (Jennings et al., 2017; Muthuramalingam et al., 2021).

The *Shigella* IpgD type III effector has been characterized in detail as a PIP_2_ 4-phosphatase involved in key processes during host cell infection (Payrastre et al., 2012). IpgD-mediated effects have been linked to its PIP_2_ hydrolytic activity, decreased inositol(1, 4, 5) triphosphate (InsP_3_) levels and increased production of PI5P (Payrastre et al., 2012). In a recent study, IpgD has been shown- similar to the *Salmonella* T3SS ortholog SopB to act as a phosphotransferase producing PI3,4P_2_ questioning the precise enzymatic activity pertinent to host cell process subversion (Walpole et al., 2022). IpgD modulates local signals occurring at bacterial-host cell contact sites during invasion and during rupture of the phagocytic vacuole by regulating the activity of small GTPases and specific phosphoinositide levels associated with the BCV (López-Montero and Enninga, 2018; Morioka et al., 2018). It also regulates Ca^2+^ signaling by depleting the second messenger InsP_3_ from T3SS-targeted cells (Sun et al., 2017) Furthermore, IpgD is the main inducer of macropinosomes surrounding invading *Shigella* (Weiner et al., 2016; Chang et al., 2020). The kinetics of (i) invasion and (ii) vacuolar rupture are tightly linked, suggesting an interdependence between both processes (Enninga et al., 2005). Due to the overall complexity of the process, it remains poorly understood how IpgD mechanistically contributes to vacuolar rupture to promote *Shigella* intracellular replication. Additional T3SS effectors, such as IpgB1/2, IpaJ, VirB and IcsB, also modulate the dynamics of host cell membranes during bacterial entry (Handa et al., 2007; Hachani et al., 2008; Heindl et al., 2010; Burnaevskiy et al., 2013; Kühn et al., 2020).

IpgD thus exhibits multiple effects on phosphoinositides as well as on Ca^2+^ signaling. Here, we review the diverse processes targeted by IpgD with a particular emphasis on findings implicating IpgD in the diversion of macropinosome formation and endosomal trafficking leading to rupture of the bacterial containing vacuole.

## Global IpgD-mediated effects on cell signaling and inflammation

**Figure 1:**
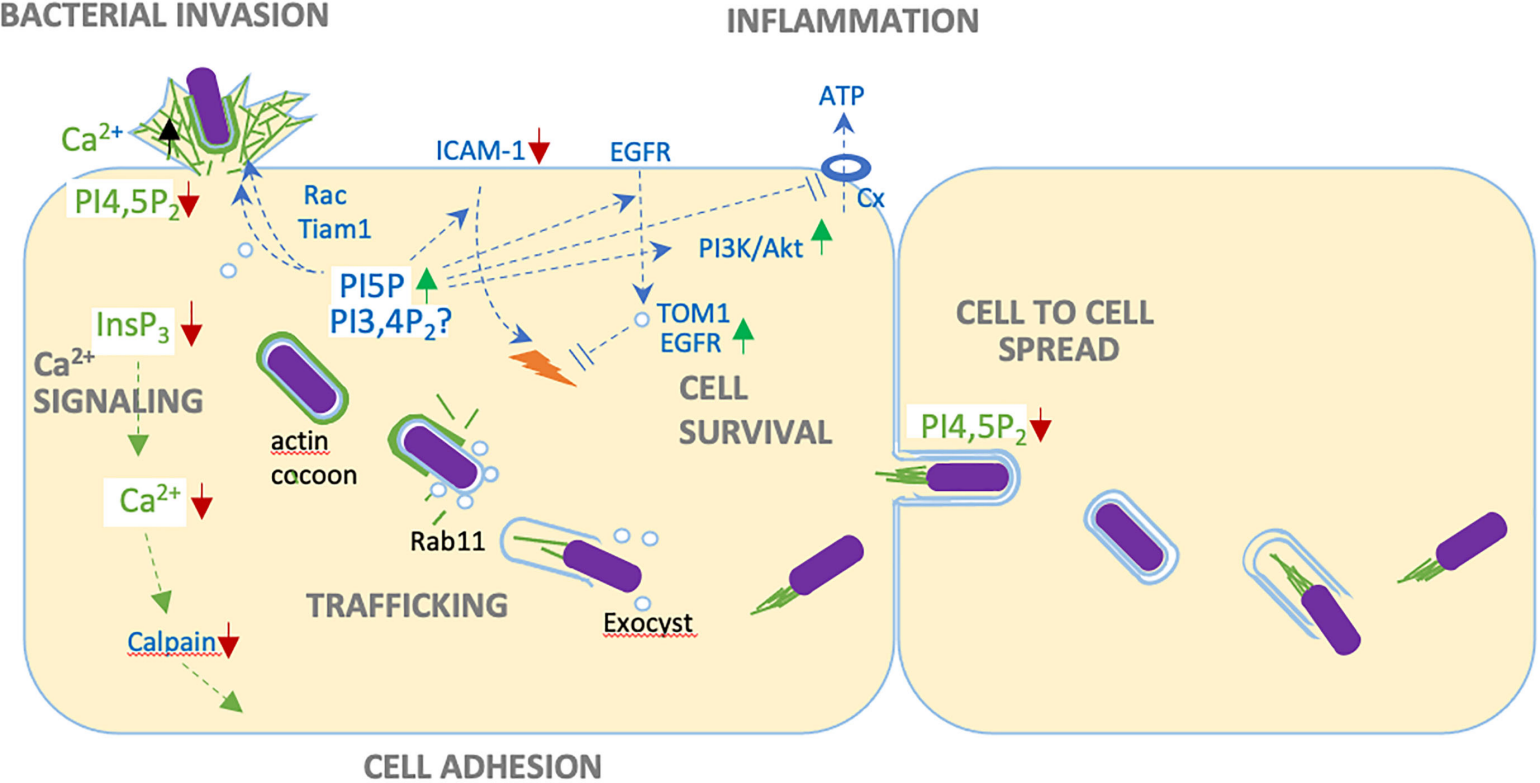
Versatile roles of IpgD during epithelial cell infection. Through PI4,5P_2_ (PIP_2_) hydrolysis, IpgD disconnect cortical actin from plasma membranes, favoring bacterial invasion and cell to cell spread. IpgD-mediated production of PI5P stimulates actin polymerization *via* Tiam-Rac activation, down-regulates the recruitment of immune cells by inducing ICAM-1 degradation, prevents the opening of connexin hemichannels, promotes persistent EGFR signaling by recruiting the TOM1 adaptor to endosome and preventing their maturation, and activates cell survival through the PI3K/Akt pathway. Possibly, these effects could also be linked to increased PI3,4P2 levels associated with the IpgD phosphotransferase activity. By decreasing InsP_3_ levels, IpgD allows long lasting local Ca^2+^ responses during invasion and prevents InsP_3_-dependent global Ca^2+^ responses at later infection stages. Inhibition of Ca2+ signaling and calpain activity favors adhesion of infected cells. Through mechanisms the remain to be characterized, IpgD stimulates the formation of macropinosomes during bacterial invasion. IpgD also allows the recruitment of Rab11-positive endosomes and the exocyst, triggering bacterial unsheathing from the actin cocoon surrounding the BCV and vacuolar escape.

Through its enzymatic activities, IpgD regulates many aspects of signaling involved in survival pathways and inflammatory processes. IpgD leads to the production of PI5P at *Shigella* invasion sites inducing the tyrosine kinase-dependent activation of class IA PI3K and activation of the Akt serine/threonine kinase regulating cell survival and proliferation (Pendaries et al., 2006) (**Figure 1**). In light of the recently described IpgD phosphotransferase activity, it is possible that Akt activation also occurs directly *via* increased PI 3,4P_2_ levels triggered by IpgD (Walpole et al., 2022). Contributing to cell survival, IpgD specifically affects the dynamics of membranes at the host cell surface including endosome and macropinosome formation. Through increased PI5P levels at the plasma membrane of infected cells and a yet to be described mechanism, IpgD stimulates endocytosis of the EGF receptor (EGFR) and the cell adhesion molecule ICAM-1 (Boal et al., 2016) (**Figure 1**). While internalization is increased for these cell surface proteins, their outcome differs suggesting differential and specific cargo sorting for EGFR and ICAM-1 endosomes (Ramel et al., 2011; Boal et al., 2016). IpgD prevents the degradation of EGFR by blocking endosome maturation through the PI5P-dependent recruitment of the TOM1 adaptor protein at endosomal membranes (Boal et al., 2015). This results in the persistence of EGFR-early endosomes and persistent EGFR-signaling favoring the PI3K-Akt survival pathway (Boal et al., 2015) (**Figure 1**). The precise mechanism involved in the PI5P/TOM1-dependent blockade of endosome maturation remains to be characterized and appears unrelated to its described function in endosomal sorting. In contrast, as expected from canonical endosomal maturation, the increased internalization of ICAM-1 leads to its targeting to lysosomes and degradation (**Figure 1**). As a global result, IpgD stimulates the removal of ICAM-1 from the epithelial cell surface, thereby reducing neutrophil adhesion and clearance of infected cells (Boal et al., 2016). Through PI5P, IpgD also prevents the recruitment of immune cells and pro-inflammatory signals by limiting the release of extracellular ATP (eATP) from infected cells (Puhar et al., 2013) (**Figure 1**). Under basal conditions the eATP concentration is negligible, while being estimated in the order of 5-10 mM in the cytosol of host cells. Therefore, eATP is a well-characterized danger signal associated with cell injury, activation of pro-inflammatory cytokines and immune cell recruitment. Bacteria, such as *Shigella*, expressing a T3SS induce the release of eATP at the early stages of infection in the absence of detectable host cell lysis (Puhar et al., 2013). *Shigella*-induced eATP release results from an increase of cytosolic Ca^2+^ levels in infected cells leading to the opening of connexin hemichannels (Puhar et al., 2013). PI5P produced by IpgD prevents connexin hemichannel opening and the release of eATP associated with *Shigella* invasion, thereby dampening associated immune responses (Puhar et al., 2013).

Finally, *Shigella* negatively regulates the recruitment of neutrophils at infection sites through direct bacterial contact with the immune cells (Konradt et al., 2011). While initially described during intestinal epithelial infection, it was also reported that injection of T3SS effectors occurred in neutrophils by transiently interacting bacteria in the absence of bacterial invasion (Konradt et al., 2011). Neutrophil migration requires dynamic reorganization of the actin cytoskeleton, as well as the membrane association of ERM family proteins acting as actin cytoskeletal linkers the hyaluronan receptor CD44 adhesion molecule. Critically, ERM protein association with the plasma membranes requires PIP_2_ (Konradt et al., 2011). Through PIP_2_ hydrolysis, IpgD prevents the membrane association of ERM proteins and neutrophil migration.

## IpgD as a key regulator of global and local Ca^2+^ signals

Because PI4,5P_2_ is the main substrate utilized by PLCs to generate InsP_3_, IpgD also depresses InsP_3_ levels by decreasing PI4,5P_2_ levels. InsP_3_ is the key second messenger leading to Ca^2+^ release (Sun et al., 2017) (**Figure 1**). During prolonged infection kinetics, IpgD virtually abolishes cytosolic Ca^2+^ increase linked to release from internal stores. The inhibition of InsP_3_-mediated signaling is expected to have versatile implications during infection. Notably, cytosolic Ca^2+^ increase is required for the activation of numerous Ca^2+^ proteases (**Figure 1**). As a result of its inhibitory effects on Ca^2+^ signaling, IpgD delays the Ca^2+^-dependent activation of calpains and disassembly of cell adhesion structures, thereby preserving the integrity of *Shigella*-infected cells (Sun et al., 2017).

Beyond inhibition, IpgD also has modulatory effects on *Shigella*-induced Ca^2+^ signals at the early stages of bacterial invasion. Shortly upon contact with host cells, *Shigella* induces atypical Ca^2+^ microdomains required for actin polymerization at invasion sites (Tran Van Nhieu et al., 2013). These *Shigella*-induced Ca^2+^ microdomains correspond to local Ca^2+^ increase, which duration reaching up to several seconds depend on the dense actin meshwork triggered by bacteria at entry sites and localized aspects of PLC activation and InsP_3_ production at sites of bacteria-host cell membranes contact (Tran Van Nhieu et al., 2013). By limiting InsP_3_ production, IpgD favors the formation of Ca^2+^ microdomains at invasion sites, while limiting the elicitation of global Ca^2+^ responses during the early stages of *Shigella* invasion (Sun et al., 2017).

## Localized action of IpgD- stimulating actin polymerization during *Shigella* entry

During contact between *Shigella* and target epithelial cells, ruffles are formed locally in the near surrounding of the bacteria. While bacterial invasion was initially described to occur through macropinocytosis, advanced imaging showed that macropinosome formation and *Shigella* entry are two distinct processes (Weiner et al., 2016). Ruffling that is required for the generation of macropinosomes is driven by the Arp2/3-dependent polymerization of actin occurring downstream of activation of the small GTPase Rac (Mounier et al., 1999; Duménil et al., 2000). Two *Shigella* type III effectors have been implicated in Rac activation and actin polymerization at invasion sites. The type III effector IpgB1 is a RacGEF amplifying actin polymerization and ruffle formation at a distance from the bacterial contact-site. The translocon component IpaC triggers actin polymerization at the intimate bacterial contact site by triggering the recruitment of the Src tyrosine kinase, to form an actin coat structure reminiscent of phagocytic cups in macrophages (Valencia-Gallardo et al., 2015). The mechanistic links between Src and Rac activation remain to be characterized and could involve phospholipases C and Ca^2+^ signaling (Tran Van Nhieu et al., 2013).

The IpgD PIP_2_ phosphatase activity favors actin polymerization at bacterial invasion sites. Seminal studies attributed this observation to the hydrolysis of PIP_2_ leading to disassembly of cortical actin and stimulating *de novo* actin polymerization in close proximity to invading *Shigella* (Niebuhr et al., 2002). It became clear, however, that IpgD could regulate actin polymerization at invasion sites in various non-exclusive manners. Through PI5P production, IpgD may lead to the recruitment of the Rac GEF Tiam1 and Rac activation at membrane contacting bacteria (Viaud et al., 2014) (**Figure 1**). Binding of PI5P to Tiam-1 stimulates its intrinsic GEF activity and Rac activation in ruffles and endosomes (Viaud et al., 2014). Also, IpgD was shown to promote the recruitment of the Arf6 GEF ARNO, likely *via* an increase of PI(3,4,5)P_3_ levels insuring a feed-back loop of Arf6 activation required for efficient invasion (Garza-Mayers et al., 2015) (**Figure 1**). While this was not investigated for *Shigella*, ARNO and Arf6 may potentiate actin polymerization in concert with the Rac-dependent recruitment of the Abi/WAVE complex as described for the *Salmonella* SopB ortholog (Humphreys et al., 2012; 2013). Another interaction partner of IpgD could be the BAR domain protein TOCA-1 that also interacts with other *Shigella* effectors; nevertheless, the implication of this interaction requires more investigation (Miller et al., 2018).

## IpgD-mediated regulation of actin after host cell entry

Upon completion of the bacterial invasion process, a thick, so-called “cocoon” forms *de novo* around the BCV (Kühn et al., 2020) (**Figure 1**) This actin cocoon represents a unique structure implicating various cytoskeletal proteins and actin nucleation by the Arp2/3 complex. It is possible to speculate that through its Nε-fatty acetylase activity the T3SS effector IcsB is required for persistent activity of the Arp2/3 complex at the actin cocoon, while the initial recruitment of this actin nucleating complex depends on the activation of the Cdc42 small GTPase, at the onset of bacterial invasion (Liu et al., 2018). The role of the actin cocoon needs to be further investigated, however evidence shows that it is required for the efficient removal of damaged vacuolar remnants following rupture, that would otherwise impair bacterial spreading. Also, the cocoon may prevent recognition of the BCV by the cell autonomous immune system. Intriguingly, IpgD appears also involved in actin cocoon regulation, however it remains to be investigated how IpgD-mediated actin polymerization/depolymerization functions are spatio-temporally regulated.

Following vacuolar escape, *Shigella* replicates freely in the cell cytosol and disseminate from cell-to-cell. Cell-to-cell spreading is permitted by the bacterial ability to use actin-based motility, pushing bacteria-containing protrusion into adjacent cells (Weddle and Agaisse, 2018) (**Figure 1**). Upon lysis of the protrusion’s double membrane, *Shigella* re-iterates replication and cell-to-cell spreading thereby disseminating over large area of the epithelial layer. Key to this process, engulfment of the bacteria-containing protrusions requires the disassembly of actin filaments at the protrusion’s membranes. IpgD was shown to contribute to the down-regulation of actin structures in these protrusions, thus favoring their resolution into double-membrane containing secondary BCVs preceding bacterial escape into the cytosol within neighboring cells and bacteria dissemination (**Figure 1**). This highlights the diverse roles of IpgD during successive steps of *Shigella* invasion (Köseoğlu et al., 2022).

## IpgD requirement during the events of vacuolar rupture

The BCV that englobes *Shigella* during its uptake differs from the macropinocytic vesicles in its close proximity, reinforcing differences between *Shigella* invasion and canonical macropinocytosis (Mellouk et al., 2014; Weiner et al., 2016; Chang et al., 2021). While bacteria invasion triggers the formation of ruffles and exocytic vesicles, the nascent BCV is formed in tight apposition with the bacterial body. The duality of macropinosome and BCV formation has been discovered through large volume correlative light and electron microscopy (CLEM) studies (Weiner et al., 2016; Weiner and Enninga, 2019). The newly formed macropinosomes cluster around the BCV within minutes upon bacterial invasion, however fusion between both compartments could not be observed. Furthermore, breaching of the BCV membrane takes place precisely at the time when it contacts macropinosomes. Macropinosomes-BCV contact sites can be visualized by 3D ultrastructural techniques, such as focused ion beam scanning EM. Studies on the formation of both compartments indicate that IpgD plays a main role in efficient vacuolar disruption (Mellouk et al., 2014; Chang et al., 2020). *ipgD* mutants show similar internalization kinetics as WT *Shigella*, however, the usually observed massive damage of the BCV is delayed. The differences between simple BCV damage and the unwrapping of the damaged BCV leaflets can be measured combining two microscopic assays; fluorescently tagged galectins that get recruited to the inner leaflet of the BCV upon its damage show the morphology of BCV disassembly, while a sensitive enzymatic FRET reporter provides precise information on the initial damage of BCV damage. Two clear defects in relation to vacuolar rupture can be attributed to the *ipgD* mutant *Shigella* strain: i, the absence of macropinosomes at bacterial entry sites; ii, the incapacity of invading *Shigella* to strip off BCV remnants that are typically transported several micrometers away upon BCV rupture. Potentially, the bacteria entrapped in broken BCVs are readily targeted by the xenophagy machinery. During these steps, forming macropinosomes become coated with Rab11, Rab8 and the exocyst complex (Chang et al., 2020), as shown by proteomics analysis of macropinosomes isolated from epithelial cells infected with *Shigella* (Chang et al., 2020; Stévenin et al., 2021) (**Figure 1**). The resuting Rab/exocyst coating is required for targeting these compartments to the BCV before its rupture. Upon Rab11 knockdown, depletion of key constituents of the exocyst complex, such as Exo70, or by using interfering peptides that hamper exocyst complex formation, the BCV and macropinosomes form less contacts. Also, damaged BCV membranes remain in close apposition to the bacteria (Mellouk et al., 2014; Chang et al., 2020), impairing intra- and inter-cellular bacterial spread as observed for a *Shigella ipgD* mutant. Whether IpgD acts only on the surrounding macropinosomes, on the BCV, or simultaneously on both compartments will require further investigation.

## IpgD orthologs: The role of SopB during *Salmonella* invasion

**Figure 2:**
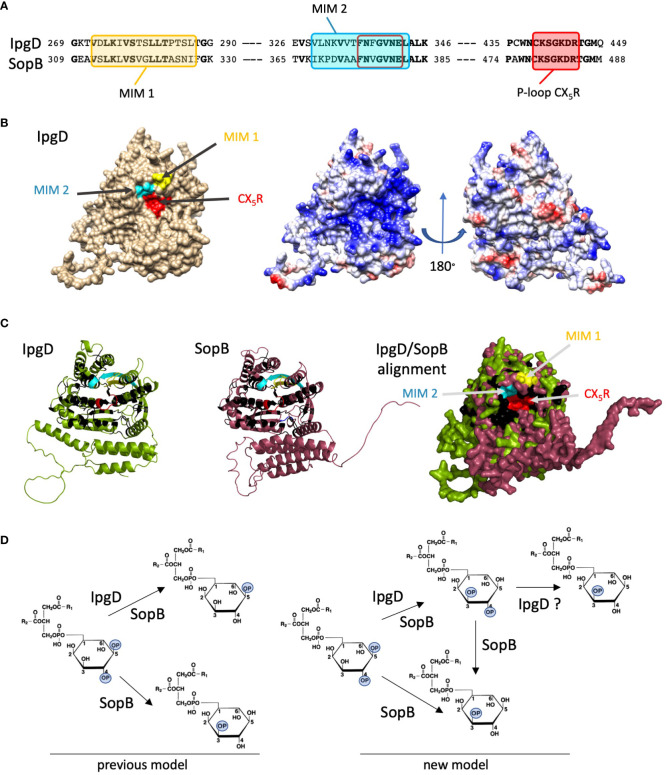
Conserved phosphoinositide phosphatase features of *Shigella* IpgD and *Salmonella* SopB. **(A)** Sequence alignments of MIMs and P-loop motives in IpgD and SopB. Bold characters: conserved residues. Yellow box: MIM1, blue box: MIM2, red box: P-loop. The MIMs were identified experimentally and through computational learning, approaches (see text). **(B)** Left: Alphafold-based *in silico* 3D structure analysis of IpgD (Jumper et al., 2021) followed by PyMOL molecular visualization (Schrödinger and DeLano 2020) showing the P-loop and surrounding MIMs. Right: surface charge distribution. Blue: positive charges, red: negative charges. **(C)** High structural homologies between IpgD and SopB. Green: IpgD, purple: SopB, yellow: MIM1, cyan: MIM2, red: P-loop, black: conserved aminoacids. Left and Middle: Alphafold-based 3D structures of IpgD and SopB. Right: overlaid 3D structures showing their high homology. **(D)** Enzymatic reactions of IpgD and SopB. PI(4,5)P_2_ dephosphorylation either yields PI5P for both enzymes or PI3P in the case of SopB (left). The recently described phosphotransferase activity leads to PI(3,4)P_2_ production that could be further dephosphorylated in PI3P (right) (Walpole et al., 2022).

*Salmonella* SopB (or SigD) was already shown to be an ortholog of IpgD more than two decades ago and it was further characterized as PI phosphatase (Norris et al., 1998). Both proteins exhibit an unstructured N-terminus likely involved in chaperone-binding and secretion by the T3SS, as well as a P-loop containing a Cys-X5-Arg motif that is a signature of the active site of Mg^2+^-independent phosphatases (Norris et al., 1998; Niebuhr et al., 2002) (**Figure 2A**, red box). In addition, a discrete hydrophobic region involved in membrane interaction has been described for SopB and suggested for IpgD (Patel et al., 2009; **Figure 2A**, yellow box MIM1. Using *in silico* computational methods for predicting topology of transmembranes 3D structures (DAS-TMpred and DeepEMPred), we confirmed MIM1 and identified a longer MIM2 for IpgD (blue box) (Cserzö et al., 1997; Hsu and Mao, 2015; Weigele et al., 2017; Wang et al., 2022). We also predicted a similar MIM2 in SopB through its sequence and structural concordances (**Figure 2A**, blue box MIM2), that includes a motif conserved in host cell phosphatases formerly identified by Norris et al., 1998. (**Figure 2A**; brown empty box)). Using Pymol, we generated an *in silico* model of IpgD that shows the spatial proximity of both MIMs to the P-loop (**Figure 2B**, left). This organization is similar to that of host phosphoinositides phosphatases, where MIMs are observed in close proximity to the active site and are presumed to facilitate accessibility to the phosphoinositide following membrane insertion (Hsu and Mao, 2015). Further stressing similarities with host lipid phosphatases, the IpgD surface surrounding the catalytic P-loop region shows a high density of positive charges likely involved in interaction with the negatively charged membranes embedding the phosphoinositide substrate (**Figure 2B**). Alphafold structure simulations of IpgD and SopB demonstrate their high levels of homology with an identical organization of MIMs and conserved residues surrounding the P-loop region (**Figure 2C**, yellow, cyan and red). Together, this analysis also shows conserved structural features and domain organization between IpgD, SopB and phosphoinosited phosphatases (Hsu and Mao, 2015). Functionally however, while *in vitro*, IpgD preferentially targets for PI(4,5)P_2,_ SopB shows preferential activity towards PI(3,4)P_2._ How the *in vitro* SopB phosphatase activity towards PI(3,4)P_2_ relates to the increased PI(3,4)P_2_ levels associated with the SopB phosphotransferase activity has remained unclear until recently, when SopB was identified as phosphotransferase similar to IpgD (Walpole et al., 2022) (**Figure 2**). During host cell challenge with *Salmonella*, SopB has been found to increase PI3P levels, which sets it apart from the production of PI5P in the case of IpgD (Hernandez et al., 2004). As for IpgD, SopB activates Akt but SopB-mediated Akt activation occurs through a process that requires PI(3,4)P_2_ and is Wortmannin insensitive (Steele-Mortimer et al., 2000; Cooper et al., 2011). Local targeting of SopB at the onset of these events has been shown to depend on myosin 6 tethering to the entry site *via* SopE (Brooks et al., 2017). Akt activation promotes intracellular bacterial growth, modulates the immune response, enhances M cell growth within the intestinal epithelium, and potentially plays a role in infection associated carcinoma (Knodler et al., 2005; Kum et al., 2011; Tahoun et al., 2012; Scanu et al., 2015). More recently, SopB has also been linked with the intracellular niche formation of *Salmonella* during epithelial infection (Stévenin et al., 2021). SopB reprograms phosphoinositides at plasma membranes of *Salmonella* invasion sites (Hernandez et al., 2004; Piscatelli et al., 2016). Furthermore, SopB plays a major role in extracting membranes from the BCV during the early invasion phase through the formation of SNX3-positive tubules (Bujny et al., 2008; Braun et al., 2010).

Contrasting with *Shigella* invasion, *Salmonella*-induced macropinosomes can fuse with the BCV, a process that is SopB -independent. SopB appears rather involved in regulating the stability of the BCV, however through the regulation of membrane influx and efflux to and from the BCV (Stévenin et al., 2019). Therefore, despite the similarities of the enzymatic function of the *Shigella* and *Salmonella* effectors IpgD and SopB, their mechanistic involvement in controlling the intracellular niche is distinct for both pathogens, perhaps due to differences in the composition of the macropinosomes forming around the respective BCVs.

## Perspectives

The function of IpgD during *Shigella* has been studied for more than two decades and has revealed its crucial involvement during entry, intracellular niche formation, dissemination as well as host immune signaling. More generally, this reflects the importance of phosphoinositide reprogramming during the course of *Shigella* infection. Phosphoinositides are key components linking membrane remodeling, cytoskeletal rearrangements, and complex signaling pathways including Ca^2+^. Based on recent discoveries reviewed here, more work is required to obtain a comprehensive understanding about the role of IpgD for *Shigella* infection. It will be particularly interesting to integrate the IpgD phosphotransferase activity with regards to its largely described phosphatase activity. As PI(3,4)P_2_ and PI(5)P trigger different signals, it will be important to decipher what is the relevance for each of the different activities during cell infection by *Shigella*. A comprehensive analysis of the similarities and differences between the *Shigella* effector IpgD and SopB of *Salmonella* will also be useful to understand the differences of their respective intracellular lifestyles. In this regard, a detailed structure-function analysis targeting specific domains of these exciting effectors will be highly informative. Novel in silico approaches, such as Alphafold are powerful tools to achieve this (**Figure 2**). Also, as IpgD acts locally at the entry site as well as globally within targeted cells, including at sites of cell-to-cell spread, it will be important to better understand the spatiotemporal organization of the action of IpgD. In this context, it would be intriguing to investigate whether IpgD gets post-translationally modified upon translocation into host cells as has been suggested for SopB (Patel et al., 2009; Popa et al., 2016). Finally, model tissues through organ-on-a-chip devices or co-cultures (Grassart et al., 2019; Mitchell et al., 2020; Rey et al., 2020) may provide with important insights to confirm the role of IpgD in *Shigella* pathophysiology.

## Author contributions

GTVN, JE, and PL-L contributed to conception and design of the study. GTVN, JE wrote the first draft of the manuscript. PL-L organized 3D structures and analysis. GTVN, JE, PL-L wrote sections of the manuscript. All authors contributed to manuscript revision, read, and approved the submitted version.

## Funding

JE and GTVN are funded by the Agence National pour la Recherche Projects “PureMagRupture” (JE, GTVN) and “RabReprogram” (JE).

## Acknowledgments

JE acknowledges support from the Institut Pasteur and the European Research Council (ERC-CoG “Endosubvert”). JE is member of the LabEx IBEID and Milieu Interieur. GTVN is supported by the Inserm (Institut National de la Santé et de la Recherche Mé dicale) and the CNRS (Centre National de la Recherche Scientifique)

## Conflict of interest

The authors declare that the research was conducted in the absence of any commercial or financial relationships that could be construed as a potential conflict of interest.

## Publisher’s note

All claims expressed in this article are solely those of the authors and do not necessarily represent those of their affiliated organizations, or those of the publisher, the editors and the reviewers. Any product that may be evaluated in this article, or claim that may be made by its manufacturer, is not guaranteed or endorsed by the publisher.
